# Survey of nasal carriage of Staphylococcus aureus and intestinal parasites among food handlers working at Gondar University, Northwest Ethiopia

**DOI:** 10.1186/1471-2458-12-837

**Published:** 2012-10-02

**Authors:** Mulat Dagnew, Moges Tiruneh, Feleke Moges, Zinaye Tekeste

**Affiliations:** 1 Department of Microbiology, School of Biomedical and Laboratory Sciences, College of Medicine and Health Sciences, Gondar University, Gondar, Ethiopia; 2 Department of Parasitology, School of Biomedical and Laboratory Sciences, College of Medicine and Health Sciences, Gondar University, Gondar, Ethiopia

**Keywords:** Food handlers, *S. aureus*, Intestinal parasites

## Abstract

**Background:**

Food borne disease are major health problems in developing countries like Ethiopia. Food handlers with poor personal hygiene working in food establishments could be potential sources of disease due to pathogenic organisms. However; information on disease prevalence among food handlers working in University of Gondar cafeterias are very scarce. The aim of this study is to assess the prevalence of nasal carriage of *Staphylococcus aureus*, their drug resistance pattern and prevalence of intestinal parasites among food handlers working in University of Gondar student’s cafeterias.

**Method:**

A cross sectional study was conducted among food handlers working in University of Gondar student’s cafeterias. A pretested structured questionnaire was used for collecting data. Nasal swab and stool were investigated for *S. aureus* and intestinal parasites; respectively as per the standard of the laboratory methods.

**Results:**

Among 200 food handlers, females comprised 171(85.5%). The majority (67.5%) of the food-handlers were young adults aged 18–39 years. One hundred ninety four (97%) of the food handlers were not certified as a food handler. Forty one (20.5%) food handlers were positive for nasal carriage of S. aureus, of these 4(9.8%) was resistant for methicilin. *Giardia lamblia* was the most prevalent parasites 22 (11%), followed by *Ascaris lumbricoides* 13(6.5%), *Entamoeba histolytica* 12 (6%), *Strongyloides stercolaris* (0.5), *Taenia species* 1(0.5%) and *Schistosoma mansoni* 1(0.5%).

**Conclusion:**

The finding stressed that food handlers with different pathogenic micro organisms may pose significant risk on the consumers. Higher officials should implement food handler’s training on food safety, periodic medical checkup and continuous monitoring of personal hygiene of food handlers.

## Background

Food borne diseases are major health problems in developed and developing countries. The World Health Organization (WHO) estimated that in developed countries, up to 30% of the population suffer from food borne diseases each year, whereas in developing countries up to 2 million deaths are estimated per year [1,2].

The spread of food borne diseases via food handlers are a common and persistent problem worldwide [3,4]. Many diseases are communicable and caused by micro-organisms that enter into the body via food [5]. Numerous outbreaks of gastroenteritis have been associated with ingestion of raw foods, foods incorporating raw ingredients or foods obtained from unsafe sources [6,7].

Food poisoning has been reported to be a result of infection with enterotoxigenic strains of *staphylococcus aureus*[8-13]. It accounts for 14–20% of outbreaks involving contaminated food in the USA [14], and in the United Kingdom restaurants are the second most important place for acquiring staphylococcal food poisoning [15]. This organism may exist on food handler’s nose or skin, from which it may be transmitted to cooked moist protein-rich foods, and become intoxication agent, if these foods are then kept for several hours without refrigeration or stored in containers.

Antibiotic resistant staphylococci are major public health concern since the bacteria can be easily circulated in the environment. Infections due to methicilin-resistant *S. aureus* (MRSA) have increased world-wide during the past twenty years [16,17]. Multiple drug-resistant *S. aureus* have been frequently recovered from foodstuffs [18], nasal mucosa of humans [19].

Likewise intestinal parasitic infections remain important public health problems in developing countries. Infection of intestinal parasites usually occurs primarily by ingestion of eggs and cysts via a fecal-oral route or directly from human to human through poor personal hygiene [20,21]. In Ethiopia amoebiasis and giardiasis are common causes of intestinal protozoa infections throughout the nation. The prevalence of amoebiasis ranges from 0–4% and that of giardiasis is 3–23% [22]. Food-handlers with poor personal hygiene working in food-serving establishments could be potential sources of infections of many intestinal helminthes, protozoa, and entero pathogenic bacteria [23]. Food-handlers who harbour and excrete intestinal parasites may contaminate foods from their faeces via their fingers, then to food processing, and finally to healthy individuals [21].

Though there are no or few indicative studies in hospital and university food catering service regarding food safety in the study area. There is no doubt food borne illnesses resulted from improper food handling. Therefore; this study aimed at assessing prevalence of nasal carriage of *S. aureus*, its drug resistance pattern and prevalence of intestinal parasites among food handlers.

## Methods

### Study design and area

A cross sectional study was conducted among food handlers working in University of Gondar students cafeterias from January 1, 2011 to June 30, 2011. Gondar town is one of the tourist destinations in Northwest Ethiopia 739 km away from Addis Ababa.

### Study population

All food handlers working in University of Gondar student cafeterias.

### Inclusion and exclusion criteria

#### Inclusion

Food handlers working in the University of Gondar student cafeterias and given informed consent were included in the study.

#### Exclusion criteria

Food handlers who had taken antibiotics and antihelminthics within the three weeks prior to the study were excluded.

### Sample size and sampling procedure

All food handlers working in University of Gondar student cafeterias namely in Gondar College of Medicine and Health Sciences, Maraki campus and Tewodros campus cafeteria. Two hundred food handlers were included in the study.

### Data collection procedure and sample collection

A pretested structured questionnaire was used for collecting information on age, sex, marital status, service years, educational status, status of training and habits of hand washing of each food-handler. Nasal swab was collected aseptically from food handlers’ nostrils rolling six times by applicator stick tipped with cotton and moistened with normal saline. Stool specimen was collected from food handlers by leak proof plastic stool cup.

#### Culture and identification

A single nasal swab was obtained from each food handler inoculated onto Manitol salt agar (MSA) and Blood agar plate (BAP) incubated for 24 hours in 35–37°C in incubator. Isolates were identified as *S. aureus* by growth characteristics on blood agar plate, MSA, Gram stain and biochemical test such as catalase test and slide coagulase were done following standard procedures [24].

### Antimicrobial susceptibility testing

Susceptibility testing was performed on Muller Hinton agar (Oxoid, Hampshire, UK ) using agar disc diffusion technique recommended by Bauer *et al.*[25]. The drugs that were tested include methicilin (10 μg), penicillin (10 μg), erythromycin (15 μg), ampicilin (30 μg), ciprofloxacin (10 μg), tetracycline (30 μg), cotrimoxazole (25 μg), and vancomycin (30 μg) (Oxoid, UK). *Staphylococcus aureus ATCC* 25922 was used as a quality control organism for the antimicrobial susceptibility test. The resistance and sensitivity were interpreted according to the National Committee for Clinical Laboratory Standards [24].

### Microscopic examination of stool

Intestinal parasites were investigated microscopically from each stool samples using both direct smears mount in saline and formol-ether concentration sedimentation procedures as per the standards [24].

### Data processing and analysis

Statistical analysis was done using SPSS version 16.00 soft ware. The chi-square test was employed to assess the association between variables. A p-value of less than 0.05 was considered to indicate statistical significance.

### Ethical consideration

The data were collected after written informed consent obtained from all study participants, and the study was approved by the Research Ethics Committee of the University of Gondar. Study participants found positive for intestinal parasites were treated and MRSA carriers were decolonized.

## Results

### Sociodemographic characterstics

A total of two hundred food-handlers, (171 of females and 29 males) were included in the study. Their mean age were 34.54 years, ranging from 18–64 years. The majority 135 (67.5%) of the food handlers were young adults aged 18–39 years. Only 105 (52.5%) of the food-handlers had education above primary school. The educational levels, age category, sex and work experiences were shown in (Table 1).

**Table 1:** Sociodemographic characteristics of food handlers working at University of Gondar in students cafeterias from January 1 to June 30, 2011

**Characteristics**	**Total**	**CMHS**	**Tewodros**	**Maraki**
		**N = 70**	**N = 75**	**N = 55**
**Age in years**	No.(%)	N0.(%)	No.(%)	No.(%)
18-28	85(42.5)	25(35.7)	31(41.3)	29(52.7)
29-39	50(25)	20(28.6)	21(28.0)	9(16.4)
40-49	31(15.5)	10(14.2)	11(14.7)	10(18.2)
50-59	31(15.5)	14(20.1)	11(14.7)	6(10.9)
60+	3(1.5)	1(1.4)	1(1.3)	1(1.8)
**Sex**				
Female	171(85.5)	63(90)	68(90.7)	40(72.7)
Male	29(14.5)	7(10)	7(9.3)	15(27.3)
**Education**				
Illiterate	11(5.5)	10(14.3)	1(1.3)	0
Grade 1-8	84(42)	31(44.3)	31(40)	22(40)
Grade 9-12	86(43)	23(32.9)	33(44)	30(54.5)
Certificate	19(9.5)	6(8.6)	10(13.3)	3(5.5)
**Service Years**				
<1 years	24(12)	8(11.4)	10(13.3)	6(10.9)
1-10 years	146(73)	48(68.6)	55(73.3)	43(78.2)
11-20 years	12(6)	6(8.6)	4(5.3)	2(3.6)
21+	18(9)	8(11.4)	6(8.1)	4(7.3)
**Total**	**200(100)**	**70(100)**	**75(100)**	**55(100)**

CMHS = College of Medicine and Health Sciences.

In hand washing practices, 179 (89.5%) food handlers had a habit of hand washing after toilet while 21(10.5%) of food handlers had no habit of hand washing after toilet. While 148 (74%) of food handlers had the habit of hand washing with soap and water, the rest 52(26% ) did not use soap for their hand after toilet. However, 92(46%) food handlers had a habit of hand washing after touching nose between handling of food items. Almost half of food handlers 93(46.5%) had no medical check-up previously including stool examination. Only 6(3%) the 200 of food handlers were certified for training in food handling and preparation (Table 2).

**Table 2:** Hygienic practice of food handlers working at the university of Gondar students cafeterias from January 1 to June 30, 2011

**Variables**	**No.(%)**
**Certified in food preparation and handling**	
**Yes**	6(3)
**No**	194(97)
**Medical check up**	
**Yes**	107(53.5)
**No**	93(46.5)
**Hand washing after using toilet by water**	
**Yes**	179(89.5)
**No**	21(10.5)
**Hand washing after using toilet with soap and water**	
**Yes**	148(74)
**No**	52(26)
**Hand washing after touching nose**	
**Yes**	92(46)
**No**	108(54)
**Hand washing before preparing food**	
**Yes**	188(94)
**No**	12(6)
**Total**	**200(100)**

### Sociodemographic in relation to carriage of *S. aureus* and intestinal parasites

In this study the rate of colonization of *S. aureus* related to age greater than 60 was 100%. However, infection to parasite age greater than 60 was 0%. The lowest rate of colonization of *S. aureus* was 15.8% in educational status of certificate. The amount of service years <1 yrs observed the lowest rate of colonization by *S. aureus* which was 8.3%. Though there were no significance association between sociodemographic variables and carriage of *S. aureus* and intestinal parasite infection (Table 3).

**Table 3:** **Sociodemographic characteristics in relation to *S. aureus *and intestinal parasites detected in food handlers at University of Gondar students cafeterias from January 1 to June 30, 2011**

**Characteristics**	**S. aureus**		**Asso.**	**Intestinal parasites**		**Association**
**Age in years**	**Negative**	**Positive**	**X**^**2**^**&P**	**Negative**	**Positive**	**X**^**2**^**&P value**
	**n(%)**	**n(%)**	**value**	**n(%)**	**n(%)**	
18-28	69(81.2)	16(18.8)		70(82.4)	15(17.6)	
29-39	37(74)	13(26)	X^2^ = 1.898	36(72)	14(28)	X^2^ = 5.119
40-49	25(80.6)	6(19.4)	P = 0.754	22(71)	9(29)	P = 0.225
50-59	25(80.6)	6(19.6)		19(61.3)	12(38.7)	
60+	0	3(100)		3(100)	0	
**Sex**						
Female	134(78.4)	37(21.6)	X^2^ = 0.936	128(74.9)	43(25.1)	X^2^ = 0.013
Male	25(86.2)	4(13.8)	P = 0.333	22(75.9)	7(24.1)	P = 0.908
**Education**						
Illiterate	9(81.8)	2(18.2)		8(72.7)	3(27.3)	
Grade 1-8	63(75)	21(25)	X^2=^3.528	57 (67.9)	27(32.1)	X^2^ = 3.87
Grade 9-12	71(82.6)	15(17.4)	P = 0.474	71(82.6)	15(17.4)	P = 0.107
Certificate	16(84.2)	3(15.8)		14(73.7)	5(26.3)	
**Year of service**						
<1 years	22(91.7)	2(8.3)		20(83.3)	4(16.7)	
1-10 years	118(80.8)	28(19.2)	X^2^ = 5.093	108(74)	38(26)	X^2^ = 4.09
11-20 years	4(33.3)	8(66.7)	P = 0.165	9 (75)	3(25)	P = 0.323
21+	15(83.3)	3(16.7)		13(72.2)	5(27.8)	
**Total**	200			200		

X^2^ = chi-square.

There is no significance association between certified in food preparation training and the presence of intestinal parasites (P = 0.810). However; there is significance association between poor hand washing practice after toilet with soap and water and the presence of intestinal parasites (P = 0.001) (Table 4).

**Table 4:** **Hygienic practice of food handlers in relation to positivity of nasal carriage of *S. aureus *and intestinal parasites at the University of Gondar students cafeteria from January 1 to June 30, 2011**

**Variables**	***S. aureus*n(%)**		**Association**	**Intestinal Parasite**		**Association**
				**n(%)**		
**Certified in food training**	Negative	Positive	X^2^ and P value	Negative	Positive	X^2^ and P value
Yes	5(83.3)	1(16.7)	X^2^ = 0 .056	4(66.7)	2(33.3)	X^2^ = 2.987
No	154(79.4)	40(20.6)	P = 0.813	46(75.3)	48(24.7)	P = 0.810
**Hand washing after toilet by water**	Negative	Positive		Negative	Positive	
Yes	144(80.4)	35(19.6)	X^2^ = 0 .938	135(75.4)	44(24.6)	X^2^ = 2.686
No	15(72.4)	6(28.6)	P = 0.333	15(72.4)	6(28.6)	P = 0.847
**Hand washing after toilet with soap**	Negative	Positive		Negative	Positive	
Yes	119(81.4)	29(19.6)	X^2^ = .286	122(82.4)	26(17.6)	X^2^ = 24.024
No	40(76.9)	12(23.1)	P = 0.593	28(53.8)	24(46.2)	P = 0.001
**Medical check up**						
Yes	81(75.7)	26(24.3)	X^2^ = 2.778	81(75.7)	26(24.3)	X^2^ = 7.038
No	78(83.9)	15(16.1)	P = 0.249	69(74.2)	24(25.8)	P = 0.855
**Hand washing before preparing food**	Negative	Positive		Negative	Positive	
Yes	150(79.8)	38(20.2)	X^2^ = 0 .159	141(75)	47(25)	X^2^ = 1.510
No	9(75)	3(25)	P = 0.690	9(75)	3(25)	P = 0.959

### Nasal carriage of *S. aureus*

Among the 200 healthy food handlers, the overall prevalence of nasal carriage of *S. aureus* was 41(20.5%). Considering the drug susceptibility pattern, all isolates of *S. aureus* were sensitive to vancomycin. However, half of the isolates of *S. aureus* 21(51.2%) and 19(46.3%) were resistant to penicillin and ampicillin; respectively. Sixteen (39%) of the isolates were resistant to amoxicillin. Thirteen (31.7%) and 11 (26.8%) of the isolates were resistant to tetracycline and cotrimoxazole; respectively. Six (14.6%) of the isolates were resistant to erythromycin, whilst 4 (9.8%) of the isolates were resistant to methicillin and ciprofloxacin; respectively (Table 5).

**Table 5:** **Antimicrobial resistance pattern of 41 *S. aureus *isolated from 200 nasal swab cultures of food handlers at the University of Gondar students cafeterias from January 1 to June 30, 2011**

**Antimicrobial agents**	**Total-resistance**	**CMHS n = 18**	**Tewodros n = 15**	**Maraki n = 8**
	**n(%)**	**n(%)**	**n(%)**	**n(%)**
Vancomycin	0	0	0	0
Methicilin	4(9.8%)	2(11)	1(6.7)	1(12.5)
Ciprofloxacin	4(9.8%)	1(5.6)	2(13)	1(12.5)
Penicillin	21 (51.2%)	9(50)	10(66.7)	2(25)
Ampicilin	19(46.3%)	8(44.4)	9(50)	2(25)
Amoxicillin	16(39%)	8(44.4)	6(40)	2(25)
Erythromycin	6(14.6%)	3(16.7)	2(13)	1(12.5)
Tetracycline	13(31.7%)	6(33.3)	5(33.3)	2(25)
Cotrimoxazole	11(26.8%)	5(27.7)	5(33.3)	1(12.5)

### Intestinal parasites

Direct microscopic and concentration techniques were used for identifying intestinal parasites from 200 stool specimens. The consistency of stool was 168(84%) formed, 18(9%) semi formed, 12(6%) diarrhea and 2(1%) dysentery. Only the formed stool was done by sedimentation concentration techniques. Fifty (25%) stool specimens were positive for different intestinal parasites. *Giardia lamblia* was the most prevalent parasites 22(11%), followed by *Ascaris lumbricoides* 13(6.5%) and *Entamoeba histolytica/dispar* 12(6%). In our study trophozoites of *G. lamblia*, *E. histolytica and* larvae of *S. stercolaris* were found in diarrhea stool. As noted in the (Table 6), *G. lamblia* and *E. histolytica*, cyst forms of the parasites are higher than the trophozoite form.

**Table 6:** Prevalence of intestinal parasites and their frequency isolated from 200 food handlers at University of Gondar students cafeterias from January 1 to June 30, 2011

**Parasite species**	**Number**	**%**
**Protozoa**		
***Giardia lamblia***	22	11
Trophozoite form	4	2
Cyst form	18	9
***Entamoeba histolytica/dispar***	12	6
Trophozoite form	3	1.5
Cyst form	9	4.5
**Helminthes**		
*Ascaris lumbricoides*	13	6.5
*Strongloid stercolaris*	1	0 .5
*Schistosoma mansoni*	1	0.5
Taenia species	1	0.5
**Total**	**50**	**25**

## Discussion

In this study, nasal swab culture and stool microscopic examination of 200 food handlers had been investigated for the presence of bacteria and intestinal parasites. The rate of isolation of *S. aureus* from the nasal cultures in our study 41 (20.5%) was found to be similar to those reported by several researchers as 26.6%, 23.1% and 21.6% [26-29]. However, our finding was found to be higher than the rate 69(0.77%) obtained from a study conducted in Turkey [30] and much lower than the findings reported in Brazil and Botswana as 30%, and 44.6%; respectively [31,32]. Nasal carriage rates reported by several workers vary and the variation has been attributed to the ecological differences of the study population.

It is very important to note that although *S. aureus* causes severe infections it may also be as a member of the normal flora of the nasal cavity [33]. If by chance, a food handlers carries, an enterotoxin producer *S. aureus* he/she may contaminate the food and causes staphylococcal food poisoning outbreak in the students population. However, in our nasal carriage strains isolated from food handlers, we were not able to identify the presence or the absence enterotoxin producer strains because of lack of reagent enterotoxin kit, Phage typing and PCR techniques.

Our study demonstrated that 4(9.8%) strains of *S. aureus* were resistant to methicillin. It is important to note that the emergence and dissemination of MRSA (Methicillin Resistant Staphylococcus aureus) is an increasing global health problem that complicates the therapeutic management of staphylococcal infections. However, the rate of resistance in our study was much lower than that reported as 3(20%) the study done in Gondar from nasal swab isolate of health professionals (unpublished). The possible explanation for the higher rate of MRSA in the previous study may be due to cross transmission with hospital strains. In this study isolates of *S. aureus* resistant to ampicilin was 19(46.3%) in line with reported as 45% in Brazil [27]. However, the resistance of *S. aureus* to penicillin in our study was lower than from the reported 70% in Brazil [31]. In our study all isolates were sensitive to vancomycin in line with a finding by Acco *et al.*[31]. However, a study conducted in Botswana showed that 9(27.3%) of the isolates were resistant to vancomycin [32].

In this study, the overall prevalence of intestinal parasite among food handlers were 50(25%) consistent with the study done in Gondar town (29%) and in Sudan 23.1% [29,34]. However, this prevalence was much lower compared to previous study done at Bahir Dar town [34], reported as 158(41.1%) and in Jimma which was 59(58.4%) [35]. The possible explanation may be more than half of the food handlers in this study had taken medical examination and might be treated for intestinal parasites or this study did not use sensitive techniques like Kato-thick smear for most of intestinal helminthes especially for *Schistosoma mansoni*, water emergency technique for *Strongloides stercolaris* and the adhesive scotch tape for *E. vermicularis.*

*A. lumbricoides, S. mansoni, Taenia species and S. stercolaris* were reported in this study, note that this parasites are not food borne pathogens. However, the presence of such pathogens may indicate low personal hygiene in food handlers and as the same time these pathogens must be treated.

It was noted that 12 (6%) and 2(1%) of food handlers working in the kitchens were suffering from diarrhea and dysentery; respectively. Active trophozoites forms of *E. histolytica*, *G. lamblia* and larva of *S. stercolaris* were associated with diarrheic food handlers. Infections with the protozoan parasites like *E. histolytica* and *G. lamblia* are common causes of diarrhoea worldwide [35]. *G. lamblia* and *E. histolytica* infected food handlers can directly transmit to consumers if ingested via contaminated food and water because *G. lamblia* cysts and *E. histolytica* cyst do not need environmental maturation. Thus, food handlers should be in a good health and those suffering from diarrhea and dysentery must be excluded from work until they have been completely free of symptoms and must get rest.

In this study, majority of food handlers working in the cafeterias were young adults 135 (67.5%) but which was older than study done in Bahir Dar 371(96.6%) [37]. More than half of (53.5%) the food handlers had medical check-up in the past. However, none of the food handlers had medical check-up in the past in Bahir Dar study [34].

## Conclusion

Multiple antimicrobial resistant strains of *S. aureus* were isolated and protozoan cysts were detected from food handlers working at University of Gondar students’ cafeterias. These findings indicate that the food handlers may be potential source of food borne disease for the students’ population being served in three cafeterias.

## Abbreviations

BAP: Blood agar plate; FEC: Formol ether concentration; MRSA: Methicillin resistant *Staphylococcus aureus*; MSA: Manitol salt agar; PCR: Polymerase chain reaction; SOPs: Standard operating procedures; WHO: World Health Organization.

## Competing interests

The authors declare that they have no competing interests.

## Authors’ contributions

MD was the primary researcher, conceived the study, designed, participated in data collection, conducted data analysis, drafted and finalized the manuscript for publication. MT and FM assisted in data collection and reviewed the initial and final drafts of the manuscript. MD, MT, FM and ZT interpreted the results, and reviewed the initial and final drafts of the manuscript. All authors read and approved the final manuscript.

## Pre-publication history

The pre-publication history for this paper can be accessed here:

http://www.biomedcentral.com/1471-2458/12/837/prepub

## References

[B1] World Health Organization Food safety and food borne illness 2007 Geneva: WHO

[B2] World Health Organization Food Safety – Food borne diseases and value chain management for food safety “Forging links between Agriculture and Health” CGIAR on Agriculture and Health Meeting in WHO/HQ 2007

[B3] Zain M Naing N Sociodemographic characteristics of food handlers and their knowledge, attitude and practice towards food sanitation: a preliminary report Southeast Asian J Trop Med Public Health 2002 33 410 417 12236444

[B4] Scott E Food safety and food borne diseases in 21^st^ century homes Can J Infect Dis 2003 14 277 280 18159469 10.1155/2003/363984 PMC2094945

[B5] Essex-caster AJ A synopsis of public health and social medicine 1987 2 Bristol: Sohn Wright, Sons Ltd

[B6] Lengerich EJ Addis DG Juranek DD Severe giardiasis in the United States J Clin Dis 1994 18 760 763 10.1093/clinids/18.5.760 8075266

[B7] Hopkins RS Juranek DD Acute giardiasis: an improved clinical case definition for epidemiologic studies Am J Epidemiol 1991 133 402 407 1994703 10.1093/oxfordjournals.aje.a115894

[B8] Carmo LS Dias RS Linardi VR Sena MJ Dos Santos DA An outbreak of staphylococcal food poisoning in the municipality of Passos, Minas Gerais, Brazil Braz Arch Biol Technol 2003 46 581 586

[B9] Arbuthnott JP Coleman DC Azavedo JC Staphylococcal toxins in human disease J Appl Bacteriol 1990 69 101 107 10.1111/j.1365-2672.1990.tb02917.x 2119059

[B10] Buckle KA Davey JA Eyles MJ Hocking AD Newton KG Stuttard EJ Food borne microorganisms of public health Significance 1993 Sydney: JM Sydney Executive Printing Service Pty Ltd 271 284

[B11] Mossel DA Netten P *Staphylococcus aureus* and related staphylococci in foods: ecology, proliferation, toxinogenesis, control and monitoring J Appl Bacteriol 1990 19 123 145 10.1111/j.1365-2672.1990.tb01804.x 2119061

[B12] Tranter HS Brehm RD The detection and aetiological significance of staphylococcal enterotoxins J Rev Med Microbiol 1994 5 56 64 10.1097/00013542-199401000-00008

[B13] Todd ECD Food borne disease in six countries. A comparison J Food Protect 1998 4 559 565 10.4315/0362-028X-41.7.559 30795101

[B14] Ughes JM Tauxe RV Mandell GL, Douglas RG, Bennett JE Food borne diseases Principles and Practice of Infectious Diseases 1990 3 New York: Churchill Livingstone 879

[B15] Wieneke AA Roberts D Gilbert RJ Staphylococcal food poisoning in the United Kingdom, 1969–90 J Epidemiol Infect Dis 1993 110 519 531 10.1017/S0950268800050949 PMC2272286 8519317

[B16] Ippolito G Leone S Lauria FN FN Nicastri E Wenzel RP Methicillin-resistant *Staphylococcus aureus:* The superbug Int J Infect Dis 2010 14 4 S7 S11 20851011 10.1016/j.ijid.2010.05.003

[B17] Deresinski S Methicillin-resistant *Staphylococcus aureus:* an evolutionary, epidemiologic and therapeutic odyssey Clin Infect Dis 2005 40 562 573 10.1086/427701 15712079

[B18] Abulreesh HH Organji SR The prevalence of multidrug-resistant staphylococci in food and the environment of Makkah, Saudi Arabia Res J Microbiol 2011 6 6 510 523 10.3923/jm.2011.510.523

[B19] Acco M Ferreira FS Henriques JAP Tondo EC Identification of multiple strains of Staphylococcus aureus colonizing nasal mucosa of food handlers Food Microbiol 2003 20 489 493 10.1016/S0740-0020(03)00049-2

[B20] Ukoli FMA Introduction to parasitology in tropical Africa 1990 New York: John Wiley and Sons 201 315

[B21] Kaferstein F Abdussalam M Food safety in the 21^st^ century B World Health Organ 1999 77 4 347 351 PMC2557642 10327714

[B22] Haile G Jirra C Mola T Intestinal parasitism among Jiren elementary and junior secondary school students, southwest Ethiopia Ethiop J Heal Dev 1994 8 37 41

[B23] World Health Organization Health surveillance and management procedures of food-handling personnel 1999 Geneva: World Health Organization 7 36 Technical report series no. 785 2505454

[B24] National Committee for Clinical Laboratory Standards Methods for determining bactericidal activity of antimicrobial agents 1993 Villanova, PA: Tentative Guidelines, M26-TNCCLS

[B25] Bauer AW Kirby WM Sherris JC Turch M Antibiotic susceptibility testing by standard single disk method Am J Clin Pathol 1966 45 493 496 5325707

[B26] Bustan MA Udo EE Chugh TD Nasal carriage of enterotoxin-producing Staphylococcus aureus among restaurant workers in Kuwait City J Epidemiol Infect 1996 116 319 322 10.1017/S0950268800052638 PMC2271425 8666076

[B27] Simsek Z Koruk I Copur A Cicek G Gulcan MD The prevalence of *Staphylococcus aureus* and intestinal parasites among food handlers in Saniurfa, Southeastern Anatolia J Public Health Manag Pract 2009 15 6 518 523 19823157 10.1097/PHH.0b013e3181aa2814

[B28] Andargie G Kassu A Moges F Tiruneh M Huruy K Prevalence of bacteria and intestinal parasites among food-handlers in Gondar Town, Northwest Ethiopia J Health Popul Nutr 2008 26 4 445 451 10.3329/jhpn.v26i4.1887 PMC2740691 19069624

[B29] Ahmed H Hassan H Bacteriological and parasitological assessment of food handlers in the Omdurman area of Sudan J Microbiol Immunol 2010 43 1 70 73 10.1016/S1684-1182(10)60010-2 20434126

[B30] Gunduz T Limoncu ME Cumen S Ari A Etiz S Tay Z Prevalence of intestinal parasites and nasal carriage of *Staphylococcus areus* among food handlers in Manisa Turky J Environ Health 2008 18 5 230 235 18561571

[B31] Acco M Ferreira FS Henriques JAP Tondo EC Identification of multiple strains of *Staphylococcus aureus* colonizing the nasal mucosa of food handlers J Food Microbiol 2003 20 5 489 493 10.1016/S0740-0020(03)00049-2

[B32] Loeto D Matsheka ML Gashe BA Enterotoxigenic and antibiotic resistance determination of *Staphylococcus aureus* strains isolated from food handlers in Gaborone, Botswana J Food Protect 2007 70 12 2764 2768 10.4315/0362-028x-70.12.2764 18095428

[B33] Williams REO Healthy carriage of staphylococcus aureus: its prevalence and importance J Bacteriol Rev 1993 27 56 71 10.1128/br.27.1.56-71.1963 PMC441169 14000926

[B34] Abera B Biadegelgen F Bezabih B Prevalence of*Salmonella typhi*and intestinal parasites among food handlers in Bahir Dar Town, Northwest Ethiopia Ethiop J Heal Dev 2010 24 1 46 50

[B35] Sahlemariam Z Mekete G Examination of fingernail contents and stool for ova, cyst and larva of intestinal parasites from food handlers working in student cafeterias in three Higher Institutions in Jimma Ethiopian J Health Sci 2001 11 2 131 137

